# Upregulation of Taurine Biosynthesis and Bile Acid Conjugation with Taurine through FXR in a Mouse Model with Human-like Bile Acid Composition

**DOI:** 10.3390/metabo13070824

**Published:** 2023-07-05

**Authors:** Teruo Miyazaki, Hajime Ueda, Tadashi Ikegami, Akira Honda

**Affiliations:** 1 Joint Research Center, Tokyo Medical University Ibaraki Medical Center, Ami 300-0395, Ibaraki, Japan; akihonda@tokyo-med.ac.jp; 2 Department of Gastroenterology and Hepatology, Tokyo Medical University Ibaraki Medical Center, Ami 300-0395, Ibaraki, Japan; h-ueda@tokyo-med.ac.jp (H.U.); ikegamit@tokyo-med.ac.jp (T.I.)

**Keywords:** taurine synthesis, bile acid conjugation, *FXR*, *Bacs*, *Baat*, *Cdo*, *Fmo1*, mouse model with human-like BA composition, *Cyp2a12*^−/−^/*Cyp2c70*^−/−^

## Abstract

Taurine, the end product in the sulfur-containing amino acid pathway, is conjugated with bile acids (BAs) in the liver. The rate-limiting enzymes in both taurine synthesis and BA conjugation may be regulated by a nucleus receptor, FXR, that promotes BA homeostasis. However, it is controversial because BAs act as natural FXR agonists or antagonists in humans and mice, respectively, due to the species differences in BA synthesis. The present study evaluated the influences of different BA compositions on both pathways in the liver by comparing *Cyp2a12*^−/−^/*Cyp2c70*^−/−^ mice with a human-like BA composition (DKO) and wild-type (WT) mice. The DKO liver contains abundant natural FXR agonistic BAs, and the taurine-conjugated BA proportion and the taurine concentration were significantly increased, while the total BA concentration was significantly decreased compared to those in the WT liver with natural FXR antagonistic BAs. The mRNA expression levels of the enzymes *Bacs* and *Baat* in BA aminations and *Cdo* and *Fmo1* in the taurine synthesis, as well as *Fxr* and its target gene, *Shp*, were significantly higher in the DKO liver than in the WT liver. The present study, using a model with a human-like BA composition in the liver, confirmed, for the first time in mice, that both the taurine synthesis and BA amidation pathways are upregulated by FXR activation.

## 1. Introduction

**Figure 1:**
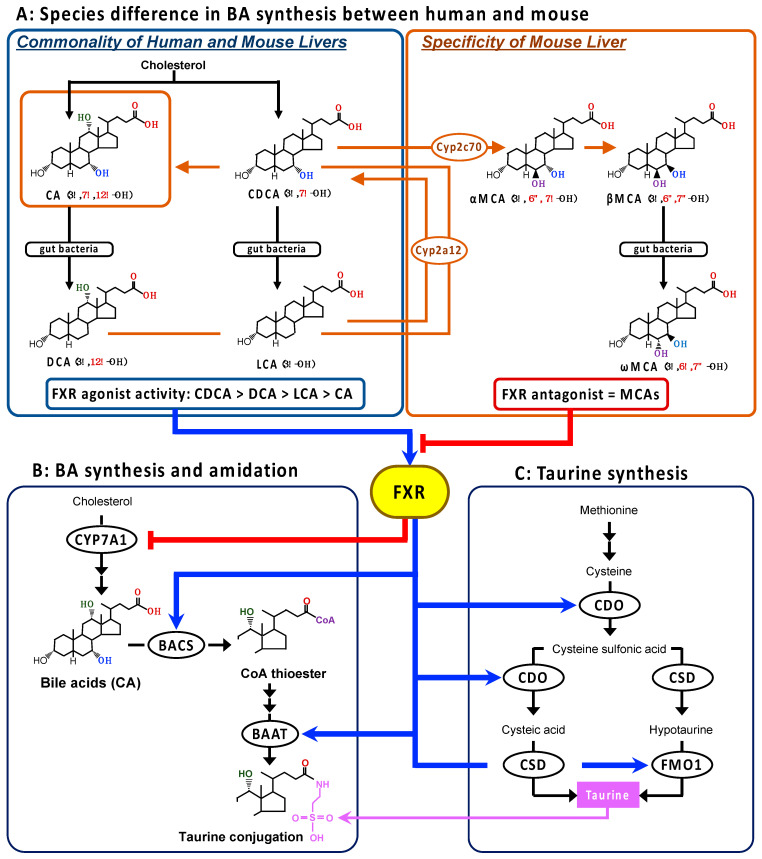
The species differences in BA synthesis in the liver between humans and mice (**A**), and the FXR regulation of the BA synthesis, BA amidation, and taurine synthesis pathways (**B**,**C**). The primary BAs, CA and CDCA, are metabolized from cholesterol and then further converted to the secondary BAs, DCA and LCA, respectively, by the intestinal bacteria in humans. In the mouse liver, CDCA is further metabolized to MCAs by Cyp2c70, and the secondary BAs are converted back to the respective primary BA by Cyp2a12. Consequently, CA and MCAs are the main BAs in mice. The mouse-specific reactions are shown in orange. The human-type BAs act as endogenous FXR agonists, and the order of ligand activity is CDCA > DCA > LCA > CA. On the other hand, MCAs behave as endogenous FXR antagonists. The gene expression of the rate-limiting enzymes in BA synthesis, BA amidation, and taurine synthesis might be regulated by FXR. Blue arrow and red line with head show the acceleration and inhibition, respectively. The orange line and the arrow show the BAs in the mouse liver and the mouse-specific metabolic pathway. Abbreviations: *BA*, bile acid; *BAAT*, bile acid-coenzyme A:amino acid *N*-acetyltransferase; *BACS*, ATP-dependent microsomal bile acid coenzyme A synthetase; *CA*, cholic acid; *CDCA*, chenodeoxycholic acid; *CDO*, cysteine dioxygenase; *CSD*, cysteine sulfinate decarboxylase; *CYP7*, CYP, cytochrome P450; *DCA*, deoxycholic acid; *FMO1*, flavin containing monooxygenase 1; *FXR*, farnesoid X receptor; *LCA*, lithocholic acid; *MCA*, muricholic acid.

Taurine (2-aminoethanesulfonic acid) is abundant in various cells and tissues in mammals. This is due to dietary intake and its biosynthesis from sulfur-containing amino acids, methionine and cysteine, which involves the rate-limiting enzymes, cysteine dioxygenase (CDO) [1] and cysteine sulfinate decarboxylase (CSD) (Figure 1C) [2,3,4,5]. Taurine has been reported to have many physiological and pharmacological roles with regard to maintaining various biological forms of homeostasis [6,7,8,9,10,11]. Among them, the most established and well-known role is the conjugation with bile acids (BAs) [12,13]. Bile acids are metabolized from cholesterol in the liver. Newly synthesized cholic acid (CA) and chenodeoxycholic acid (CDCA), the primary BAs in humans, are then conjugated at the C24 carboxyl group with taurine or glycine (Figure 1B) [14]. Through the conjugation with amino acids (amidation), the polarity of BAs is increased, and, consequently, the excretion into the bile, the formation of bile micelle, and the intestinal absorption of lipids and lipid-solved vitamins are promoted. In addition, the cytotoxicity of hydrophobic BAs is reduced [13,15]. The excreted primary BAs conjugated with taurine or glycine in the intestine are deconjugated by intestinal bacteria, and then converted to the secondary BAs, deoxycholic acid (DCA) and lithocholic acid (LCA) (from CA and CDCA, respectively), following dehydroxylation at the 7α-hydroxy group by intestinal bacteria (Figure 1A) [16]. The deconjugated BAs are conjugated again with the amino acids in the liver following intestinal absorption in the enterohepatic circulation. The BA amidation is carried out in the peroxisome and endoplasmic reticulum through sequential enzyme reactions involving two limiting enzymes: ATP-dependent microsomal BA coenzyme A (CoA) synthetase (BACS), which converts a BA to an acyl-CoA thioester; and BA-CoA:amino acid *N*-acetyltransferase (BAAT), which transfers the acyl-CoA thioester to taurine or glycine (Figure 1B) [17,18]. Almost all BAs are conjugated with taurine in rodents [19], while the ratio of taurine- and glycine-conjugation is 1:3~3.5 in humans [20,21].

Bile acids play the role of the endogenous agonist of a nuclear receptor, farnesoid X receptor (FXR; NR1H4), to regulate the transcription of associated genes in the hepatic synthesis and enterohepatic circulation of BAs to avoid BA accumulation in the liver [22,23]. It is also involved in the downregulation of microsomal cholesterol 7α-hydroxylase (*CYP7A1*), the rate-limiting enzyme in the BA synthetic pathway (Figure 1B), through the upregulation of the atypical nuclear receptor, small heterodimer partner (*SHP*; NR0B2) [24], the upregulation of ABC transporter family including the bile salt export pump (*BSEP*; ABCB11), which actively excretes BAs into the bile canaliculi [24], and the downregulation of the basolateral sodium/taurocholate co-transporter peptide (*NTCP;* SLC10A1), which transports BAs into the liver from the portal vain [24].

In addition, the rate-limiting enzyme genes in BA amidation, *BACS* and *BAAT*, have been shown to be the direct target of FXR (Figure 1B) [25,26]. Pircher et al. showed that both *BACS* and *BAAT* genes are positively regulated via inverted repeat-1 elements cognate to the FXR in human and rat hepatocytes [26]. The increased transcriptional levels of both genes were also confirmed in the liver of rats treated with the synthetic FXR agonist GW4064 (3-[2-[2-chloro4-[[3-3(2,6-dichlorophenyl)-5-(1-methylethyl)-4-isoxazolyl]methoxy]phenyl]ethenyl]benzoic acid) [26]. Therefore, it makes sense that both the rate-limiting enzymes in BA amidation and taurine synthesis are positively regulated by FXR activation, based on the role of FXR in accelerating BA excretion from the liver tissue.

On the other hand, in vivo studies using mice showed that the *Baat* and *Csd* gene expression levels were significantly reduced by GW4064 treatment, suggesting that the taurine synthetic enzymes are negatively regulated by the FXR–SHP axis [27]. Thus, there is a discrepancy in mice with regard to the responses of BA amidation and taurine synthesis to the role of FXR on BA homeostasis. A possible reason for this is the species difference, particularly between humans and mice, in BA metabolism [28]. In the liver, CA and CDCA are the end-product, as the primary BAs in humans, while CDCA is further metabolized to muricholic acids (MCAs) by Cyp2c70 in mice (Figure 1A) [28]. Moreover, the secondary BAs are converted by Cyp2a12 back to their respective primary BAs in the mouse, but not the human, liver (Figure 1A) [28]. The potency order of the FXR agonist activity among the natural BAs is CDCA > DCA > LCA > CA [29,30], but MCAs act as FXR antagonists [31]. Thus, the hepatobiliary concentrations of BAs with more potent FXR agonists are almost deficient, whereas the concentrations of natural FXR antagonistic MCAs are abundant in mice.

In order to eliminate the species difference in the BA metabolism with regard to humans, we created a mouse model with human-like BA composition by double knockouts of *Cyp2a12* and *Cyp2c70* (DKO) [28,32,33,34,35]. The purpose of this study is to evaluate the influences of alteration in the composition of human-type BAs with higher FXR agonist activity on BA amidation and taurine biosynthesis by comparing the DKO mouse to the wild-type (WT) mouse, which has endogenous FXR antagonistic MCAs.

## 2. Materials and Methods

### 2.1. Materials

Cholic acid, glycocholic acid (GCA), taurocholic acid (TCA), CDCA, glycochenodeoxycholic acid (GCDCA), taurochenodeoxycholic acid (TCDCA), DCA, glycodeoxycholic acid (GDCA), taurodeoxycholic acid (TDCA), LCA, glycolithocholic acid (GLCA), taurolithocholic acid (TLCA), ursodeoxycholic acid (UDCA), glycoursodeoxycholic acid (GUDCA), αMCA, βMCA, ωMCA, tauro-αMCA (TαMCA), tauro-βMCA (TβMCA), and tauro-ωMCA (TωMCA) were obtained from Steraloids, Inc. (Newport, RI, USA). [^2^H_4_]cholic acid, [^2^H_4_]DCA, and [^2^H_4_]LCA were purchased from C/D/N Isotopes, Inc. (Pointe-Claire, QC, Canada). [^2^H_4_]taurocholic acid was obtained from Merck, KGaA (Darmstadt, Germany). [^2^H_4_]chenodeoxycholic acid was supplied from the Research Laboratory of Nippon Kayaku Co. (Toyo, Japan). Tauroursodeoxycholic acid (TUDCA), [^2^H_4_]UDCA, and [^2^H_4_]TUDCA were supplied by Tokyo Tanabe Company (Tokyo, Japan).

3-Aminopyridyl-*N*-hydroxysuccinimidyl carbamate (APDS), acetonitrile, amino acids mixture standard solutions (Type B, AN-2), ammonium acetate, APDSTAG Wako Amino Acids Internal Standard (IS) mixture solution, APDSTAG Wako Eluent, dicalcium phosphate, ethanol, formic acid, methanol, potassium phosphate, sodium borate buffer, sucrose, and taurine were purchased from FUJIFILM Wako Pure Chemical Corporation (Osaka, Japan).

The RNeasy Plus Mini Kit was purchased from Qiagen K.K. (Tokyo, Japan). The reverse transcription using the PrimeScript RT reagent kit and the mouse housekeeping gene primer set were purchased from Takara Bio, Inc. (Shiga, Japan). The FastStart DNA Master SYBR Green I and the LightCycler system were obtained from Roche Diagnostics (Mannheim, Germany).

### 2.2. Animals

Wild-type C57BL/6J mice (male, body weight 26.9 ± 0.6 g, *n* = 8) and DKO (*Cyp2a12^−/−^* and *Cyp2c70^−/−^*; male, 28.3 ± 0.5 g, *n* = 8) mice were used according to our previous studies [28,32,33]. The mice were bred and kept until 20 weeks of age at the Jackson Laboratory Japan, Inc. (Ishioka, Japan) under a regular 12 h light–dark cycle (6:00–18:00) with regular food (CRF-1, Oriental Yeast Co., Ltd., Tokyo, Japan) and water ad libitum in pathogen-free conditions. After fasting overnight with free access to water, mice were euthanized by exsanguination under combination anesthesia with medetomidine, midazolam, and butorphanol. Liver tissue was collected and frozen at –80 °C until analysis.

### 2.3. BA Analysis

Liver tissue was homogenized with ice-cold ten-times volume of phosphate-buffered saline (PBS), and the supernatant was collected after centrifugation at 3500× *g* for 10 min at 4 °C. In the supernatant, BAs were measured using a high-performance liquid chromatography–electrospray ionization tandem mass spectrometry (HPLC-ESI-MS/MS) system according to the previous reports [28,36]. In brief, 200 µL of the supernatant was mixed with 20 µL of IS mixture (41.6 ng of [^2^H_4_]CA, 57.5 ng of [^2^H_4_]CDCA, 32.8 ng of [^2^H_4_]DCA, 22.4 ng of [^2^H_4_]LCA, 34.4 ng of [^2^H_4_]UDCA, 34.4 ng of [^2^H_4_]TUDCA, 100 ng of [^2^H_3_]TCA in acetonitrile), and 2 mL of 0.5 M potassium phosphate buffer (pH 7.4). The sample was eluted using Bond Elut C18 cartridges (200 mg; Agilent Technologies, Santa Clara, CA, USA) and was evaporated at 100 °C under a nitrogen stream. Then, the dried residue was redissolved in 20 mM ammonium acetate–methanol buffer (pH 7.5) and centrifugated at 12,000× *g* for 1 min. An aliquot of the supernatant was injected into the HPLC-ESI-MS/MS system for analysis. For further information on the extraction of BAs, see the previous study [37].

### 2.4. Amino Acid Analysis

Amino acids in the liver, including taurine, methionine, and cysteine, were measured by a derivatization method with APDS using the HPLC-ESI-MS/MS system according to the previously reported method [38,39]. In brief, 50 µL of the supernatant of liver tissue homogenized with PBS was mixed with an equal volume of APDSTAG IS solution and two-times volume of acetonitrile. After centrifugation at 20,000× *g* for 10 min, 20 µL of the supernatant was mixed with an equal volume of APDS-acetonitrile solution (20 mg/mL) and three-times volume of 0.2 M sodium borate buffer (pH 8.8), and then incubated at 55 °C for 10 min. Next, the reaction mixture was added to 100 μL of formic acid solution (0.1% in water), and 5 μL was used for injection into the HPLC-ESI-MS/MS system. For further information on the general HPLC and MS/MS conditions, see the previous studies [38,40].

### 2.5. Total RNA Extraction and RT-PCR Analysis

Fifty mg of the liver tissue was homogenized with a 10-times volume of lysis buffer, and then total RNA was extracted using the RNeasy Plus Mini Kit. Five-handled ng of total RNA was reverse-transcribed to cDNA using the PrimeScript RT reagent kit. The mRNA expressions of *Fxr*, *Shp*, *Cyp7a1*, *Cdo*, *Csd*, *Bacs*, *Baat*, *favin containing monooxygenase (Fmo) 1*, *Fmo3*, *Bsep*, *Ntcp*, and *hepatocyte nucleus factor 4α* (*Hnf4α*) were quantified with real-time quantitative PCR using gene-specific primers (Table 1) in the FastStart DNA Master SYBR Green I and the LightCycler system (Roche Diagnostics). PCR amplification began with a 10 min preincubation step at 95 °C, followed by 40 cycles of denaturation at 95 °C for 10 s, annealing at 62 °C for 10 s, and elongation at 72 °C. The relative concentration of the PCR products derived from the target gene was calculated using the LightCycler System software. A standard curve for each run was constructed by plotting the crossover point against the log concentration. The concentration of target molecules in each sample was then calculated automatically with reference to this curve (r = −1.00), and the specificity of each PCR product was assessed by melting curve analysis. The mRNA expression of each gene was standardized to the expression of *Ywhaz* as a housekeeping gene that was selected from the mouse housekeeping gene primers including *Atp5f1*, *B2m*, *Hprt1*, *Rplp1*, *Ppia*, *Rps18*, *Pgk1*, *Gusb*, *Tbp*, *Actb*, *Tfrc*, *Ywhaz*, *18SrRNA*, and *Gapdh* by geNorm and Bestkeeper algorisms (Supplementary Table S1) [41].

### 2.6. Statistical Analysis

Statistical analysis was carried out using Jump, and the threshold for significant differences was set at a *p*-value of 0.05; the significance was assessed using unpaired Student’s *t*-tests. Data are expressed as the mean ± standard error (SE).

## 3. Results

### 3.1. Bile Acid Concentrations in the Liver

**Figure 2:**
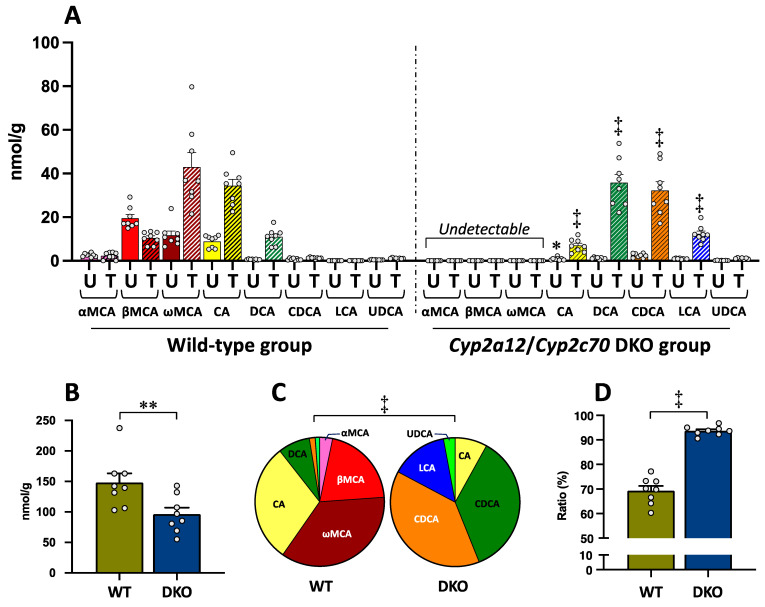
Bile acid concentrations in the liver of the WT and DKO groups. (**A**) Concentration of all BA types conjugated with and without taurine. Data are the mean ± SE. The white circle in the column shows the individual value. * *p* < 0.05, ‡ *p* < 0.0001 compared to the respective BA in the WT group by two-way ANOVA post hoc Bonferroni’s multiple comparison test. Abbreviations: *U*, unconjugated form (plane column); *T*, taurine-conjugated form (striped column). (**B**) TBA concentration (the sum of all BA types of unconjugated and taurine-conjugated forms). Data are the mean ± SE. ** *p* < 0.01 according to unpaired Student’s *t*-test. (**C**) BA composition (the sum of unconjugated and taurine-conjugated forms). Pie graph was constructed using the mean of each BA. ‡ *p* < 0.0001 according to chi-square test. (**D**) The ratio of taurine-conjugated BAs. Data are the mean ± SE. ‡ *p* < 0.0001 according to unpaired Student’s *t*-test.

Unconjugated and taurine-conjugated forms of all the BA types are shown in Figure 2A. In the WT group, MCAs (αMCA, βMCA, and ωMCA), CA, and DCA of both forms were abundantly contained in the liver, but the CDCA, DCA, LCA, and UDCA levels were so low as to be undetectable (Figure 2A). On the other hand, MCAs of both forms were undetectable, while CDCA and LCA, in addition to CA and DCA, were found in the DKO liver (Figure 2A). Ursodeoxycholic acid level was still very low in the DKO group. In the DKO group, both unconjugated and taurine-conjugated CA concentrations were significantly lower than in the WT group, while the taurine-conjugated forms of other human-type BAs, TCDCA, TDCA, and TLCA, were significantly higher compared to those in the WT group (Figure 2A). Any types of BAs conjugated with glycine were undetectable in the liver of both groups.

Total BA (TBA) concentration (the sum of all BA types with unconjugated and taurine-conjugated forms) in the liver was significantly decreased in the DKO group as compared to the WT group (Figure 2B).

Figure 2C shows the BA composition consisting of each BA type (the sum of unconjugated and taurine-conjugated forms) in the liver of both groups. There was a significant difference in the BA composition between the two groups (Figure 2C). In the WT group, the ratios of MCAs (the sum of αMCA, βMCA, and ωMCA) and human-type BAs (the sum of CA, CDCA, DCA, LCA, and UDCA) were 59.7 ± 1.6% and 40.3 ± 1.7%, respectively. On the other hand, all of the BA compositions were changed to the human-type BAs in the DKO group. Particularly, the ratios of DCA, CDCA, and LCA were 8.0 ± 0.8%, 1.3 ± 0.2%, and 0.05 ± 0.01%, respectively, in the WT group, while they were increased to 38.6 ± 0.9%, 35.7 ± 0.7%, and 14.2 ± 0.9%, respectively, instead of the disappearance of MCAs, in the DKO group (Figure 2C). The BA compositions in the DKO liver (Figure 2C) became similar to those of normal liver tissue in humans previously reported (CA 28.7%, CDCA 46.4%, DCA 15.7%, LCA 4.5%, and UDCA 4.6%) [42].

As shown in Figure 2D, the proportion of taurine-conjugated BAs (the sum of all BA types) in the liver was 69.3 ± 2.1% in the WT group; on the other hand, the proportion was significantly increased to 93.7 ± 0.7% in the DKO group.

### 3.2. Amino Acid Concentrations in the Liver

**Figure 3:**
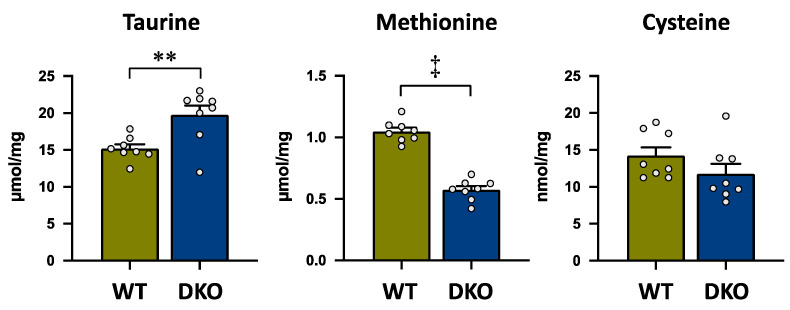
Taurine and the sulfur-containing amino acids, methionine and cysteine, which are the precursors of taurine; concentrations in the liver of both groups. The data are presented as the mean ± SE. The white circle in the column shows the individual value. ** *p* < 0.01, and ‡ *p* < 0.0001 by unpaired Student’s *t*-test.

Figure 3 shows taurine and its precursor amino acids in the biosynthesis pathway, methionine and cysteine, in the livers of both groups. Taurine concentration was significantly higher in the DKO group than in the WT group (Figure 3). On the other hand, methionine concentration was significantly lower in the DKO group than in the WT group (Figure 3). Similarly, cysteine concentration tended to be lower, but not significantly, in the DKO group than in the WT group (Figure 3).

### 3.3. The mRNA Expression Levels in the Liver

**Figure 4:**
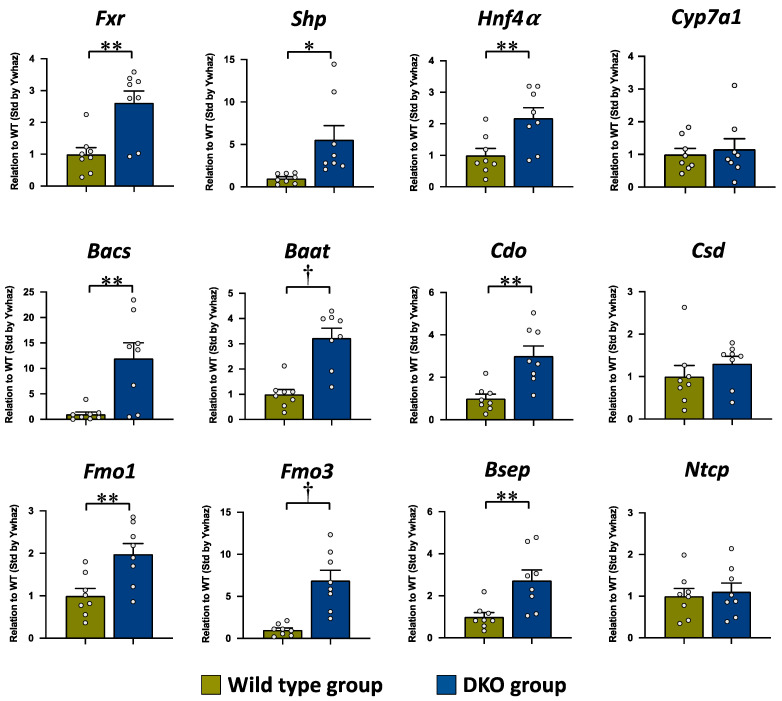
The mRNA expressions of *Fxr* and its target genes involved in BA synthesis, BA amidation, taurine synthesis, and BA transport in the liver tissue. The mRNA expression level is expressed in relation to that of the WT group, after standardization by *Ywhaz*. The data are presented as the mean ± the SE. The white circle in the column shows the individual value. * *p* < 0.05, ** *p* < 0.01, and † *p* < 0.001 according to unpaired Student’s *t*-test.

The mRNA expressions of *Fxr* and its direct target gene, *Shp*, were significantly increased in the DKO group as compared to the WT group, but there was no difference in *Cyp7a1* mRNA between both groups (Figure 4). The mRNA expressions of *Bacs* and *Baat*, the rate-limiting enzymes in BA amidation, were also significantly higher in the DKO group compared to those in the WT group. In the rate-limiting enzymes of the taurine synthesis pathway, the mRNA expression level of *Cdo* was significantly higher in the DKO group than in the WT group, while there was no difference in *Csd* mRNA expression between both groups. The mRNA expression levels of *Fmo1*, which is also a key enzyme in taurine biosynthesis [42], and *Fmo3*, which is the direct FXR target gene [43], were significantly higher in the DKO group compared to those in the WT group. In addition, the mRNA expression levels of *Hnf4α* (NR2A1), which is a nucleus receptor, and *Bsep*, which is an efflux BA transporter, were significantly higher in the DKO group than in the WT group, while there was no significant difference in *Ntcp*, which is an influx BA transporter, between both groups.

## 4. Discussion

The present study evaluated the alterations of BA amidation and taurine biosynthesis properties in the livers of the DKO mice, which have abundant FXR agonist human-type BAs, in comparison to the WT mice, which have FXR antagonistic BAs. As shown in our previous studies [28,33], the BA composition in the liver was completely changed to human-type BAs by the DKO of *Cyp2C70* and *Cyp2a12*, which are the enzymes that convert CDCA to αMCA, and the secondary BAs to the primary BAs. In the DKO liver, the ratio of CDCA and DCA, which have the most and second highest FXR agonist activity, was increased up to approximately 40%, while the WT liver was occupied by MCAs, which are the endogenous FXR antagonist, and CA, which has little activity as an FXR agonist. Upon the change to a human-like BA composition, the proportion of taurine-conjugated BA in the DKO liver reached over 90%, a 20% point increase compared to the WT liver. Furthermore, the taurine concentration was significantly increased by 25% in the DKO liver compared to the WT liver.

In the evaluation of the mRNA expression levels, the significant increase in *Shp* in the DKO liver implies that the FXR was activated by the increase in endogenous FXR agonists CDCA and DCA. This FXR activation should induce the enhancements of BA conjugation with taurine and taurine biosynthesis reactions through the upregulations of FXR target genes involving these reactions. In BA amidation, the mRNA expressions of *Bacs* and *Baat* were significantly increased in the DKO liver; particularly, the increase in the *Bacs* gene was markedly higher than the *Baat* expression. Both *Bacs* and *Baat* have been shown to be the direct target of FXR in human and rat hepatocytes, and the upregulations of them were induced in the synthetic FXR agonist GW4064-treated rat liver [26]. The present results agree with the previous observations of Pircher et al. [26]. Furthermore, the mRNA expression of *Bsep*, which is an efflux BA transporter, was significantly increased in the DKO liver. According to the increased BA-taurine conjugation ratio, along with the upregulation of mRNA expressions of the BA amidation enzymes as well as the efflux BA transporter, the significantly decreased TBA concentration in the DKO liver was highly suggestive of the enhancement of efficient biliary BA excretion from the liver. In the taurine biosynthesis pathway, the present study showed significantly increased mRNA expression levels of *Cdo*, but not *Csd,* in the DKO liver, and this can be considered to be related to the significant increase in taurine concentration in the DKO liver. From this result, the gene expression of *Cdo* is suggested to be upregulated by a direct or indirect FXR–SHP axis pathway. In the *Cyp2c70* single KO mice with higher CDCA concentration, but not in the *Cyp2a12* single KO mice with MCAs, reported in our previous study [28], the increases of BA-taurine conjugation ratio and mRNA levels of these enzymes in BA amidation and taurine synthesis were also observed in the liver, supporting the positive regulations on the reactions by FXR activation (Supplementary Figure S1).

However, the previous study reported opposite responses with regard to the gene expressions in BA–taurine conjugation and taurine biosynthesis in the WT mice treated with GW4064, i.e., the significantly decreased mRNA expression levels of *Baat* and *Csd* in the synthetic FXR-agonist-treated liver [27]. A possible reason for the opposite response to the FXR agonist in the WT mouse liver as compared to the present and previous findings [26] might be related to the balance of the endogenous FXR agonists and antagonists that were permanently present. The opposite results for these gene expressions under GW4064 treatment between mice [27] and rats [26] were likely due to the different ratios of MCA and CDCA contents in the liver, because rats do not metabolize all CDCA to MCA, and the amount of CDCA is still maintained to the MCA level in rats [44]. However, further study is needed to clarify how the different balances of endogenous FXR agonists and antagonists influence the response of gene expressions to the synthetic FXR agonist.

Unexpectedly, the *Fxr* mRNA level was significantly higher in the DKO liver than in the WT liver. Although the regulation of *Fxr* transcription has been clarified, chronic treatment of the semisynthetic FXR agonist 6α-ethyl CDCA (INT-747, obeticholic acid) has been reported to increase Fxr expression in the penile tissue of rabbits and rats [45]. Therefore, there is a possibility that *Fxr* transcription might be ligand-dependent and positively regulated. However, the increased *Fxr* mRNA expression was not found in the *Cyp2c70* single KO liver [28], and it is unclear if the *Fxr* transcriptional system in the mouse liver has a similar system in the *Fxr* transcription to the penile tissue in rabbits and rats. Thus, this point also requires more investigation.

In the DKO liver, the taurine precursor amino acids, methionine and cysteine, were lower, while taurine was higher, compared to those in the WT liver. The decreased level of these amino acids might be due to the enhanced synthesis of taurine. Recently, Veeravalli et al. reported that the subtype 1 of FMO (FMO1) is the new candidate to be the key enzyme in the taurine synthesis pathway to oxidize hypotaurine to taurine (Figure 1) [42]. In the DKO liver, *Fmo1* and another subtype, *Fmo3*, expressions were significantly higher compared to those in the WT liver. Flavin-containing monooxygenase type 3 is the direct FXR target gene and oxidizes trimethylamine (TMA), which is metabolized from choline and carnitine in the gut by intestinal bacteria, to trimethylamine oxide in the liver [46]. It is notable that methionine is the metabolic source of the methyl group donor in many metabolic pathways of trimethyl products, including choline and carnitine, and is not only used in taurine biosynthesis. Therefore, the significant decrease in methionine is likely due to the utilization of other metabolites such as choline and carnitine. Similar to Fmo3, Fmo1 is suggested to be also regulated by FXR and might contribute to the significant increase of taurine concentration in the DKO liver. However, further studies are needed to clarify the details of the regulation of Fmo1 by FXR.

The DKO mouse with the human-like BA composition is considered to be a model that extrapolates FXR regulation on BA amidation and taurine synthesis in humans. However, there is a species difference in the taurine synthesis property; rodents can highly synthesize taurine, while taurine synthesis is very low in humans [47]. If taurine biosynthesis is promoted by FXR activation, a question arises as to why taurine synthesis is high in mice with MCAs. The reason is still unclear, but there is a high possibility that the taurine synthesis pathway is mainly regulated by another uncertain mechanism, in addition FXR, under the presence of MCAs. However, it is also certain that the taurine synthesis pathway might be additionally promoted by FXR activation in liver under human-like BA compositions.

In addition, the proportion of BA amidation is different among animal species; all BAs in mice are conjugated with taurine, while the conjugation ratio with taurine and glycine is 1:3~3.5 in humans [20,21]. In humans, the reason for the low taurine conjugation ratio is considered to be due to the lower taurine pool in the liver compared to mice, and the taurine pool in humans is capable of only one quarter of the conjugation demand of BAs in the liver [21]. On the other hand, there is no species difference in the affinity of BAAT to taurine, and the affinity of BAAT in the human liver is higher with taurine than with glycine [48]. Furthermore, the ratio of conjugation with taurine is increased by taurine supplementation even in humans, while glycine supplementation does not increase the ratio conjugated with glycine [13,21]. Therefore, because the determining factor of the lower ratio of BA–taurine conjugation in humans is the lower taurine pool, the proportion of BA conjugation with taurine would be enhanced by the promotion of taurine synthesis through FXR activation. Because the conjugation with taurine increases the hydrophilicity of the BA molecule, as opposed to the conjugation with glycine [49], and because taurine itself has many beneficial effects on various tissues and cells [6,7,8,9,10,11,25], the increase in the taurine pool would lead to important physiological and pathological effects on bile excretion, cholesterol reduction, and hepatic protection. Recently, the relationship between taurine level in the body and healthy life span has been reported in the study of various animals including humans [50]; taurine abundance decreased during aging and the prevention of this decline by taurine supplementation increased health span and lifespan in the monkeys and rodents. Because the decrease of taurine synthetic ability is a high possible reason for the age-related decline of taurine pool, clarification of the regulation of taurine synthesis contributes healthy life and longevity. In the clinic, therapeutic effects for BA analogs and synthetic FXR agonists on various liver injury and metabolic disorders, including primary biliary cholangitis, nonalcoholic fatty liver disease, nonalcohol hepatitis, and lipodystrophy, have been reported [51]. Considering the present results, the enhancement of BA amidation and taurine synthesis might contribute to the therapeutic effects of these FXR agonists.

In the present and our previous studies [28], the mRNA expressions of *Cyp7a1* and *Ntcp*, which are negatively regulated by FXR, were not reduced, but the FXR–SHP pathway was activated, in the DKO liver. Competitively to the downregulations by FXR activation, the transcriptions of *Cyp7a1* [52,53,54] and *Ntcp* [52,55] are positively regulated by HNF4α, and the transactivation of *Hnf4α* is inhibited by BAs [56]. The marked increases of *Cyp7a1* and *Ntcp* expressions have been observed in the liver with decreased BAs of the patients with cerebrotendinous xanthomatosis which is the inherited deficiency of key-enzyme (sterol 27-hydroxylase) in BA synthesis, although the FXR–SHP pathway was normally activated [57]. The significantly increased *Hnf4a* expression in the DKO liver was induced concomitantly with the significant decreases of TBA in the liver and the body pool in the present and previous studies [28]. Furthermore, the significantly increased taurine concentration in the DKO liver is likely to be a factor to increase *Cyp7a1* expression, because taurine itself has the effect to increase *Cyp7a1* mRNA expression as well as its activity in rodents [58,59,60]. In addition, taurine has been reported to act as an agonist of the nucleus receptor, liver X receptor α (LXRα; NR1H3) [61], which positively regulates *Cyp7a1* expression [62,63]. In the DKO liver, we showed that the expressions of target genes for Lxrα, including *sterol regulatory element-binding protein 1* and *ATP-binding cassette transporter A1,* and the biliary proportion of cholesterol were significantly increased, suggesting the activation of Lxrα [28]. Thus, the change to human BA composition also causes a concomitant decrease in BA content and an increase in taurine content in the DKO liver. Therefore, the absence of *Cyp7a*1 and *Ntcp* expression suppression despite Fxr activity may be related to competitive regulation by activation of Hnf4α and Lxrα in the DKO liver.

## 5. Conclusions

In the present study, using a model with a human-like BA composition in the liver, we confirmed, for the first time in mice, that a high content of BAs with FXR agonist activity upregulates taurine biosynthesis in addition to BA conjugation with taurine. Because the hydrophilicity of taurine-conjugated BAs is higher than that of glycine-conjugated BAs, and because taurine itself has various beneficial actions, the promotion of taurine conjugation and biosynthesis through FXR activation is considered meaningful for human health.

## Figures and Tables

**Table 1:** Gene sequences of the PCR primers.

Gene	Accession Number		Sequence (5′-3′)	Product Size (bp)
*Bacs*	NM_009512.2	F	TCT ATG GCC TAA AGT TCA GGC G	75
		R	CTT GCC GCT CTA AAG CAT CC	
*Baat*	NM_007519	F	GTG TAG AGT TTC TCC TGA GAC AT	199
		R	CTG GGT ACA GGT GGG TAG AC	
*Bsep*	NM_021022	F	AGC AGG CTC AGC TGC ATG AC	122
		R	AAT GGC CCG AGC AAT AGC AA	
*Cdo*	NM_033037.4	F	GGG GAC GAA GTC AAC GTG G	162
		R	ACC CCA GCA CAG AAT CAT CAG	
*Csd*	NM_001359126	F	CCA GGA CGT GTT TGG GAT TGT	193
		R	ACC AGT CTT GAC ACT GTA GTG A	
*Cyp7a1*	NM_007824	F	AAG AGC AAC TAA ACA ACC TG	244
		R	TTC CCA CTT TCA TCA AGG TA	
*Fmo1*	NM_010231.3	F	CCA TCA AGT GCT GCC TGG AA	143
		R	CCT GCT GCT GTT AGA AAC CAC AGA	
*Fmo3*	NM_008030.1	F	CCA CAG CAG GGA CTA TAA GGA A	129
		R	GAG CTG ATG GTG ACC TTC TGA	
*Fxr*	NM_001163700	F	GGT CAT GCA GAC CTG TTG GAA	142
		R	TGA CGA TCG CTG TGA GCA GA	
*Hnf4α*	NM_008261.3	F	ATG CCT GCC TCA AAG CCA TC	67
		R	ATC TTG CCC GGG TCA CTC A	
*Ntcp*	NM_001177561	F	AAG GCC ACA CTA TGT ACC CTA CGT C	106
		R	GAT GCT GTT GCC CAC ATT GA	
*Shp*	NM_011850	F	CAA GGA GTA TGC GTA CCT GA	232
		R	GAT AGG GCG GAA GAA GAG AT	

*Bacs*, ATP-dependent microsomal bile acid CoA synthetase; *Baat*, bile acid-CoA:amino acid *N*-acetyltransferase; *Bsep*, bile salt export pump; *Cdo*, cysteine dioxygenase; *Csd*, cysteine sulfinate decarboxylase; *Cyp7a1*, cytochrome P450 7a1; *Fmo1/3*, flavin containing monooxygenase 1/3; *Fxr*, farnesoid X receptor; *Hnf4α*, hepatocyte nuclear factor 4α; *Ntcp**,*** sodium/taurocholate co-transporter peptide; *Shp*, small heterodimer partner; *F*, forward; *R*, reverse; *bp*, base pair.

## Data Availability

Data sharing is not applicable to this article.

## Supplementary Materials

The following supporting information can be downloaded at: https://www.mdpi.com/article/10.3390/metabo13070824/s1, Figure S1: The ratio of taurine-conjugated bile acids and the mRNA expressions of *Fxr* target genes in BA amidation and taurine synthesis in the liver of *Cyp2a12* KO and *Cyp2c70* KO mice.; Table S1: Evaluation stability and ranking of 14 candidate reference genes evaluated by geNorm and Bestkeeper algorisms.

## Abbreviations

APDS	3-Aminopyridyl-N-hydroxysuccinimidyl carbamate
BA	bile acid
BAAT	bile acid-coenzyme A:amino acid *N*-acetyltransferase
BACS	ATP-dependent microsomal bile acid coenzyme A synthetase
BSEP	bile salt export pump
CA	cholic acid
CDCA	chenodeoxycholic acid
CDO	cysteine dioxygenase
CoA	coenzyme A
CSD	cysteine sulfinate decarboxylase
CYP	cytochrome P450
DCA	deoxycholic acid
DKO	double knockout
FXR	farnesoid X receptor
FMO1/3	flavin containing monooxygenase 1/3
GCA	glycocholic acid
GCDCA	glycochenodeoxycholic acid
GDCA	glycodeoxycholic acid
GLCA	glycolithocholic acid
GUDCA	glycoursodeoxycholic acid
GW4064	3-[2-[2-chloro4-[[3-3(2,6-dichlorophenyl-5-(1-methylethyl-4-isoxazolyl]methoxy]phenyl]ethenyl]benzoic acid
HNF4α	hepatocyte nuclear factor 4α
HPLC-ESI-MS/MS	high-performance liquid chromatography–electrospray ionization tandem mass spectrometry
IS	internal standard
LCA	lithocholic acid
LXRα	liver X receptor α
MCA	muricholic acid
NTCP	sodium/taurocholate co-transporter peptide
PBS	phosphate-buffered saline
SE	standard error
SHP	small heterodimer partner
TBA	total bile acid
TCA	taurocholic acid
TCDCA	taurochenodeoxycholic acid
TDCA	taurodeoxycholic acid
TLCA	taurolithocholic acid
TUDCA	Tauroursodeoxycholic acid
TαMCA	tauro-α-muricholic acid
TβMCA	tauro-β-muricholic acid
TωMCA	tauro-ω-muricholic acid
UDCA	ursodeoxycholic acid
WT	wild type
