# Supplementary material for: Upregulation of Taurine Biosynthesis and Bile Acid Conjugation with Taurine through FXR in a Mouse Model with Human-like Bile Acid Composition
Source: Metabolites. 2023 Jul 5;13(7):824. doi: 10.3390/metabo13070824 (PMC10385265; doi:10.3390/metabo13070824)
Supplement: Supplementary file 1 [file metabolites-13-00824-s001.zip › metabolites-2424859-supplementary.pdf]

Supplementary Table S1. Evaluation stability and ranking of 14 candidate reference genes evaluated by geNorm and Bestkeeper algorisms.

Reference Gene	Accession number	geNorm M Value	geNorm Rank	BestKeeper SD	BestKeeper Coefficient of correlation	BestKeeper Rank
Ywhaz *	NM_011740	0.205	1	0.99	0.981	1
B2m *	NM_009735	0.205	1	>1	0.983	
*Hpt1**	NM_013556	0.234	3	>1	0.973	
*Tbp**	NM_013684	0.250	4	0.98	0.978	2
*Pgk1**	NM_008828	0.273	5	>1	0.984	
*Atp5f**	NM_009725	0.283	6	>1	0.976	
*Ppia**	NM_008907	0.289	7	>1	0.981	
*Tfrc**	NM_011638	0.301	8	0.96	0.963	3
*Actb**	NM_007393	0.318	9	>1	0.969	
*Gusb**	NM_010368	0.328	10	>1	0.965	
*Gapdh**	NM_008084	0.343	11	>1	0.911	
*Rps18**	NM_011296	0.364	12	>1	0.954	
*Rplp1**	NM_018853	0.407	13	>1	0.925	
18SrRNA	X56974	0.429	14	0.80	0.908	4

* Mouse Housekeeping Gene Primer Set, Takara Bio, Inc. Abbreviation of gene: *18SrRNA* , 18S ribosomal RNA; *Actb*, actin, beta; *Atp5f1*, ATP synthase peripheral stalk-membrane subunit b; *B2m*, beta-2 microglobulin; *Gapdh*,

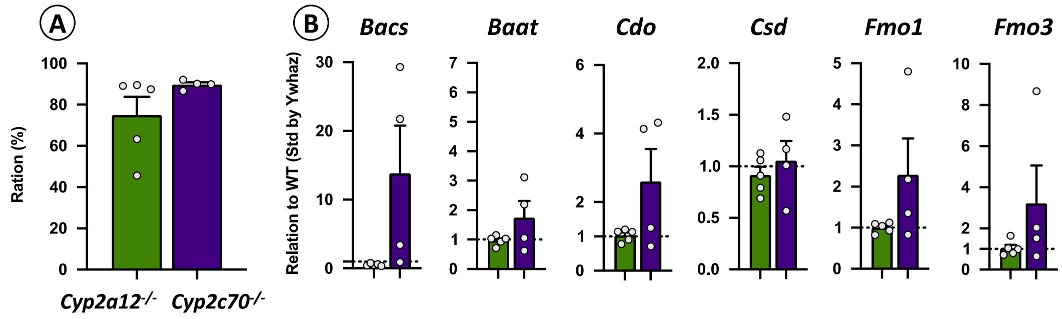

KO (n=5) and *Cyp2c70* KO (n=4) mice. The mRNA level is expressed in the relation to the WT group after standardization by Ywhaz. All data are presented as the mean ± standard error.
